# Infertility: a continually neglected component of sexual and reproductive health and rights

**DOI:** 10.2471/BLT.20.252049

**Published:** 2020-06-02

**Authors:** Jessica D Gipson, Marta J Bornstein, Michelle J Hindin

**Affiliations:** a Fielding School of Public Health, University of California, Los Angeles, United States of America (USA).; b Reproductive Health, Population Council, 1 Dag Hammarskjold Plaza New York, New York 10017, USA.

Having a child if and when desired is a fundamental component of our life as humans. The rights of individuals to found a family and decide freely and responsibly the number, spacing and timing of their children, and to have the information and means to do so feature in international human rights documents as early as 1948.^1^ These rights serve as a foundation to address the reproductive needs of populations. Efforts to address reproductive needs have predominantly focused on preventing unintended pregnancies, pregnancies that are too early, too late or too many. However, these efforts neglect to fully acknowledge and address the magnitude and consequences of infertility, that is, pregnancies that occur later than desired or do not occur at all, despite being wanted.

The need to address infertility was a central component of the 1994 International Conference on Population and Development Programme of Action, which stated that all 179 signatory countries should make the prevention and appropriate treatment of infertility accessible. Yet, in the past 25 years, little progress has been made. In the 2018 Guttmacher–*Lancet* commission on Sexual and Reproductive Health and Rights,^2^ authors noted that infertility has not been prioritized by global public health policy-makers and has received far less attention when compared to other issues within these rights. Authors also noted the scarcity of domestic and international funding to support programmatic and research efforts on infertility, particularly in low-income countries.^2^

Here we posit that with a predominant and narrow focus on the prevention of unintended pregnancy, the field of sexual and reproductive health rights (and we ourselves, as researchers in this field)^3^ have inadvertently contributed to the lack of attention to infertility, stunting efforts to address reproductive rights more holistically. We also discuss how the prevailing limited view of fertility has neglected the full range of human rights and hampered efforts to address infertility.

While rapid population growth continues to impede economic development and adversely affects the health and well-being of our global population and planet, millions of people^4^^,^^5^ are affected by infertility, often with devastating social consequences, such as abandonment and stigma.

These coexisting realities of unintended pregnancy and infertility were not considered when policy-makers designed and implemented national population policies and associated programmes in the 1960s and 1970s, nor are they considered now. Despite a shift towards a more rights-based approach in the 1990s, the legacy of population control has lasting effects on structures and policies that we must actively work to change. Preventing unintended pregnancy through ensuring the basic human right to access effective contraceptive methods is central to reproductive autonomy and to declines in fertility. However, the field’s priorities continue to be too narrowly focused, failing to embrace the fact that the need to prevent unintended pregnancy through support for contraception exists in parallel with the need to support the achievement of fertility for those who have difficulties in conceiving and in reaching desired fertility. An integrated, complementary approach to addressing unwanted pregnancy and infertility would lead to a more holistic human rights approach, and provide an opportunity to better understand each of these health issues in their own right.^6^ Perhaps most importantly, an integrated approach would reflect the reality of populations in navigating the double burden of unintended pregnancy and infertility, and of individuals who may face both unintended pregnancy and infertility during their reproductive life course.

We know little about the magnitude and consequences of infertility due to our focus on reducing unintended pregnancy and the omission of infertility in sexual and reproductive health rights research and programming. Current estimates of infertility range from 48.5 million couples^4^ to 186 million individuals^5^ worldwide. This broad range of estimates underscores the lack of reliable population-level data on infertility, knowledge of who is affected, and documentation of the full magnitude and burden of infertility and its consequences. Numerous measurement challenges impede efforts to obtain accurate and reliable estimates of infertility, including inconsistent definitions of infertility; reliance on clinic-based samples for prevalence estimates (which are not representative of the population of women and men with infertility); limitations in the design and sampling in population-based surveys;^7^ the effects of social norms, stigma and other barriers to reporting experiences of infertility and care-seeking for infertility; and the omission of men and male perspectives, among others. Despite these challenges’ magnitude, we argue that they are comparable to those that arise in the measurement of unintended pregnancy and its consequences if we prioritize infertility in the same way we have prioritized limiting fertility.

We suggest that policy-makers and sexual and reproductive health rights researchers, experts and donors adopt three actions to address this situation. First, conduct rigorous investigations to determine the magnitude of infertility and its consequences. Efforts are underway to standardize definitions of infertility^8^ and to use available nationally representative data to improve population-based estimates of infertility.^9^ However, these efforts are sparse, underfunded and have not fully addressed sampling and measurement issues,^10^ especially in contrast to other global health conditions. Dedicated and standardized field-tested measures will allow us to develop more accurate estimates of how many women and men are affected by infertility, and to identify the etiology and type of infertility to inform treatment and interventions. With these data, we can begin to quantify the full impact and costs of infertility. Second, address the neglected and growing gap in infertility programming and research. Changes in global demographic patterns, including delays in childbearing, mean the need for infertility research and interventions will increase. We need to invest in high-quality research and in generating evidence now, to address infertility and ensure basic human rights. Third, develop robust and multidisciplinary research efforts to better prevent, detect, manage and treat infertility. The successful development and implementation of infertility research and interventions require the integration of biomedical, public health and social science perspectives to address the biological and social aspects of infertility across and within populations.

Integrated efforts are necessary to combat the existing and growing inequities in access to infertility services based on social class or geography, to promote and expand access to proven and acceptable low technology and low-cost treatment options, and to mitigate the physical and social effects of infertility.^11^ Global efforts to achieve universal health coverage (UHC) provide new opportunities to address these inequities;^12^ however, the inclusion of infertility diagnosis and treatment must be part of UHC efforts. 

We must also critically assess the most appropriate, ethical, feasible and locally relevant approaches to provide infertility services (that is, to prevent and treat infertility) within existing health-care systems and social systems and to prevent any unintended consequences, particularly for marginalized populations. A key step in normalizing and addressing infertility is the provision of infertility screening and treatment within health systems.

We can no longer afford to exclude infertility as part of the global sexual and reproductive health and rights agenda. We must consider the needs of individuals and couples, understand their lives and their struggles, and identify feasible, sustainable ways to facilitate achievement of their fertility goals, whether that is wanting fewer children, or wanting more. We will better meet the sexual and reproductive needs of women and men globally by embracing a more humane and holistic perspective, and meaningfully integrating infertility into sexual and reproductive health rights programmes and empirical research.
